# New Drug Updates in Solid Tumors: PARP Inhibitors in Ovarian Cancer, Immunotherapeutics, and Other Agents

**Published:** 2018-04-01

**Authors:** Edward Li

**Affiliations:** University of New England College of Pharmacy, Portland, Maine

## Abstract

Therapies approved for solid tumors by the FDA from 2016 to 2017 have transformed the way clinicians practice medicine. Dr. Li from the University of New England College of Pharmacy provides the latest clinical trial data and insights on the emergence of new drug classes such as PARP inhibitors and PD-1/PD-L1 inhibitors.

From late 2016 through 2017, many drug approvals in oncology represented marginal advances over existing agents. The approvals included additions to the therapeutic categories of poly (ADP-ribose) polymerase (PARP) inhibitors, cyclin-dependent kinase (CDK) 4/6 inhibitors, programmed cell death protein 1 (PD-1)/programmed cell death ligand 1 (PD-L1) inhibitors, and tyrosine kinase inhibitors (TKI), as discussed at JADPRO Live 2017 by Edward Li, PharmD, MPH, BCOP, of the University of New England College of Pharmacy in Portland, Maine. The emergence of these new drug classes has helped transform clinical practice for many types of cancer. PARP inhibitors have an established role in the management of ovarian cancer. CDK4/6 inhibitors represent the standard of care in hormone receptor (HR)–positive, human epidermal growth factor receptor 2 (HER2)–negative advanced breast cancer. PD-1/PD-L1 have an established role in multiple types of cancer, most recently urothelial carcinoma. New TKIs demonstrated activity in non–small cell lung cancer (NSCLC) refractory to existing agents and as extended adjuvant therapy in breast cancer.

"We have new products that change the way we practice medicine," said Dr. Li. "But many oncology drug approvals in the last year are what we would consider ’me-too’ types of drugs."

## PARP INHIBITORS

Approval and availability of PARP inhibitors began in 2014 with olaparib (Lynparza). The mechanistic activity of PARP inhibitors relates to the role of *BRCA* mutations in oncogenesis. BRCA proteins are essential for repair of single- and double-stranded breaks in DNA by means of a process known as homologous recombination repair (HRR). *BRCA* mutations render cells incapable of repairing the breaks, a status known as homologous recombination deficiency (HRD) or synthetic lethality. PARP inhibitors target HRD to disrupt *BRCA*-mutant cancer cell growth and proliferation (Sonnenblick, de Azambuja, Azim, & Piccart, 2015).

The PARP enzyme has functions other than DNA repair, Dr. Li continued. It has key roles in chromosomal stability, mitosis, and programmed cell death, or apoptosis. As a result, the clinical application of PARP inhibitors has evolved beyond *BRCA* mutation status and HRD.

The two newly approved PARP inhibitors are rucaparib (Rubraca; December 2016) and niraparib (Zejula; March 2017). Additionally, the FDA approved a tablet formulation of olaparib, which reduces the pill burden. Rucaparib has approval as monotherapy for *BRCA*-mutated advanced ovarian cancer treated with at least two prior chemotherapy regimens. Niraparib and olaparib have approval as maintenance therapy for recurrent, platinum-sensitive ovarian cancer.

Rucaparib received approval on the basis of the open-label phase II ARIEL2 trial (Swisher et al., 2017). Data analysis included 192 patients: 40 who had *BRCA*-mutated ovarian cancer, 82 who had *BRCA* wild-type disease and high-level loss of heterozygosity (LOH), and 70 who had *BRCA* wild-type tumors and low LOH. Median progression-free survival (PFS) was 12.8 months in the *BRCA*-mutant subgroup, 5.7 months in the LOH-high subgroup, and 5.2 months in the LOH-low group (*p* < 0.001; *p* = .011). Median duration of response was significantly longer in the *BRCA*-positive (9.2 months) and LOH-high groups (10.8 months) than in the LOH-low group (5.6 months, *p* = .013, *p* = .022).

The pivotal phase III, randomized, placebo-controlled trial of niraparib included 553 patients with platinum-sensitive recurrent ovarian cancer (Mirza et al., 2016). They were randomized 2:1 to maintenance therapy with niraparib or placebo, and the trial had a primary endpoint of PFS. The study population comprised 203 patients with germline *BRCA* mutations and 350 without.

Niraparib maintenance resulted in a median PFS of 21.5 months vs. 5.5 months with placebo in the patients with germline *BRCA* mutations (*p* < .001). The remaining 350 patients included subgroups with HRD and those without. In the HRD subgroup, niraparib and placebo resulted in a median PFS of 12.9 and 3.8 months, respectively (*p* < .001). In the non-HRD subgroup, median PFS was 9.3 months with niraparib and 3.9 months with placebo (*p* < .001). Overall survival has yet to be reported.

For comparison, olaparib received FDA approval as maintenance for recurrent, platinum-sensitive ovarian cancer on the basis of a phase II randomized, placebo-controlled trial (Ledermann et al., 2014). The study involved 265 patients with recurrent, platinum-sensitive ovarian cancer, including 136 patients with *BRCA* mutations.

Median PFS among patients with *BRCA* mutations was significantly longer with olaparib (11.2 vs. 4.3 months, *p* < .0001). In the *BRCA* wild-type subset, olaparib also resulted in longer PFS, although the difference from placebo was more modest (7.4 vs. 5.5 months, *p* = .0075).

A subsequent exploratory analysis of overall survival showed that patients randomized to olaparib had a 27% reduction in the hazard for progression or death, but the difference did not meet the prespecified level of significance (*p* = .025 vs. *p* < .0095). In the subgroup of patients with *BRCA* mutations, olaparib led to significantly longer median survival (34.9 vs. 30.2 months, *p* = .025). Survival did not differ between treatment arms for patients without *BRCA* mutations.

"We don’t really look at overall survival data all that much in a phase II study; we tend to look at that as being descriptive in nature," said Dr. Li. "So even though this looks good, we still have to interpret that with caution as to whether or not that overall survival benefit really is there."

With regard to adverse effects, bone marrow suppression is a class effect of PARP inhibitors. As such, patients should be monitored for transformation to myelodysplastic syndrome and acute myelogenous leukemia. Increased serum creatinine is specific to rucaparib, said Dr. Li. Patients treated with niraparib develop hypertension more often than do patients treated with the other two agents in the class. Pneumonitis is a particular concern with olaparib.

Niraparib and olaparib have identical indications: maintenance therapy following platinum-based chemotherapy for patients with relapsed, platinum-sensitive disease, irrespective of *BRCA* mutation status (National Comprehensive Cancer Network, 2017). Rucaparib has approval for treatment of recurrent *BRCA*-mutant ovarian cancer in patients who have received at least two prior chemotherapy regimens. Rucaparib also is preferred for patients with recurrent platinum-resistant ovarian cancer.

## CDK4/6 INHIBITORS

As with PARP inhibitors, two new members of the therapeutic class recently received FDA approval—ribociclib (Kisqali) and abemaciclib (Verzenio)—joining palbociclib (Ibrance), which was approved in early 2016, Dr. Li noted. All three drugs received approval for the treatment of advanced HR-positive, HER2-negative breast cancer.

The FDA-approved indication for ribociclib stipulates use of the drug in combination with an aromatase inhibitor as initial endocrine therapy for postmenopausal women with advanced/metastatic HR-positive/HER2-negative breast cancer. Palbociclib has the same indication.

Abemaciclib has approval for use with fulvestrant (Faslodex) in patients whose disease has progressed on endocrine therapy. Abemaciclib may also be used as a single agent to treat HR-positive/HER2-negative advanced/metastatic breast cancer that has progressed following endocrine therapy and prior chemotherapy in the metastatic setting. In February 2018, abemaciclib was approved in combination with an aromatase inhibitor in the first-line setting (FDA, 2018).

CDK4/6 help regulate signaling in the retinoblastoma (Rb) gene pathway (VanArsdale, Boshoff, Arndt, & Abraham, 2015). In the normal state, Rb functions as a tumor suppressor, but in the mutated state, Rb protein undergoes phosphorylation, which effectively turns off the growth-suppressing properties. CDK4/6 plays a major role in the inactivation of Rb. Treatment with a CDK4/6 inhibitor inactivates the kinases, leading to dephosphorylation of Rb and cell-cycle arrest, thereby preventing cancer cell growth and proliferation.

The three CDK4/6 inhibitors differ somewhat in their selectivity, which translates into differences in side-effect profiles. Palbociclib and ribociclib inhibit CDK4 and CDK6, but palbociclib inhibits the two proteins equally, whereas ribociclib has 5-fold greater selectivity for CDK4, Dr. Li explained. Abemaciclib also inhibits CDK4 and CDK6 but also CDK9 and has 9-fold greater selectivity for CDK4 as compared with CDK6.

"The more selective you are against CDK4, the less neutropenia you’ll have," said Dr. Li.

The pivotal trial for ribociclib was the phase III, randomized, double-blind, placebo-controlled MONALEESA-2 study (Hortobagyi et al., 2016). The study involved 668 postmenopausal women with recurrent/metastatic HR-positive/HER2-negative breast cancer and no prior systemic therapy in the metastatic setting. Patients received ribociclib plus the aromatase inhibitor letrozole (Femara) or placebo plus letrozole. The trial had a primary endpoint of PFS.

The trial ended after a preplanned interim analysis showed a 44% reduction in the hazard for progression or death among patients randomized to ribociclib (*p* = 3.29 × 10^-6^). The median PFS had yet to be reached with ribociclib vs. 14.7 months with letrozole alone. For indirect comparison, the pivotal trial of palbociclib yielded a median PFS of 20.2 months for patients treated with the CDK4/6 inhibitor plus letrozole vs. 10.2 months for patients who received only the aromatase inhibitor (Finn et al., 2015).

Supporting evidence for abemaciclib’s two FDA-approved indications came from separate clinical trials. The indication for last-line monotherapy was based on the single-arm MONARCH 1 trial involving 132 patients (Dickler et al., 2017) Treatment with the CDK4/6 inhibitor led to a median PFS of 6 months and a median overall survival of 17.7 months.

The indication for second-line use in combination with fulvestrant was based on data from the randomized, placebo-controlled phase III MONARCH 2 trial (Sledge et al., 2017). Patients received either the combination or fulvestrant plus placebo, and the results showed a median PFS of 16.4 months with abemaciclib and 9.3 months for fulvestrant alone (*p* < .001).

For many clinicians, differing toxicity profiles of the CDK4/6 inhibitors will determine the choice of drug for a patient. Given its longer time on the market, palbociclib provides a frame of reference, and the most common adverse events are fatigue, nausea, and diarrhea. Ribociclib has a similar profile. Fatigue affects a similar proportion of patients treated with any of the drugs in the class, but abemaciclib is associated with more gastrointestinal problems, particularly diarrhea, but also more nausea, abdominal pain, and decreased appetite.

The picture changes with laboratory abnormalities, said Dr. Li. Abemaciclib is associated with substantially less grade ≥ 3 neutropenia, as compared with ribociclib and palbociclib. Anemia and thrombocytopenia occur with similar frequency across the class, as well as febrile neutropenia. Ribociclib can lead to prolongation of the cardiac QT interval and elevated creatinine levels. Elevated serum creatinine also occurs more often with abemaciclib as compared with palbociclib, as does hypokalemia.

With respect to special monitoring, neutropenia is an issue across the class, although less of an issue with abemaciclib. Diarrhea requires special attention for patients treated with abemaciclib.

Patients on any of the CDK4/6 inhibitors should have standard monitoring for venous thromboembolism and hepatic toxicity. The association of ribociclib with QT prolongation may require special attention as well.

Noting the clinical scope of approved indications for CDK4/6 inhibitors, Dr. Li concluded, "What all of the data tell us is that a patient with HR-positive, HER2-negative breast cancer should get a CDK4/6 inhibitor somewhere along their treatment pathway. Whether or not it’s upfront, in the middle, or at the end, they’ll benefit from a CDK4/6 inhibitor."

## PD-1/PD-L1 INHIBITORS

In the first half of 2017, avelumab (Bavencio) and durvalumab (Imfinzi) became the fourth and fifth members of the PD-1/PD-L1 therapeutic class to gain FDA approval. Both drugs received indications for locally advanced or metastatic urothelial carcinoma that has progressed during or after platinum-based chemotherapy. Additionally, avelumab received approval for metastatic Merkel cell carcinoma.

PD-1/PD-L1 inhibitors are part of the broader molecular category of checkpoint inhibitors, which currently consist of the five anti–PD-1 agents and the cytotoxic T-lymphocyte–associated antigen 4 (CTLA-4) inhibitor ipilimumab (Yervoy).

Briefly reviewing the mechanistic basis of checkpoint inhibitors, Dr. Li noted that checkpoints exist to prevent the immune system from "going wild," mounting an uncontrolled attack against antigens and peripheral tissues, resulting in a disseminated inflammatory response that eventually would prove to be fatal. Checkpoints help maintain immune system homeostasis.

CTLA-4 has broader activity as compared with the PD-1/PD-L1 checkpoints, which operate more selectively in peripheral tissues (Pardoll, 2012). Dr. Li likened the difference to house lighting: turning lights on and off in the entire house vs. a specific room. PD-1/PD-L1 signaling downregulates T-cell activity. Inhibitors of the signaling pathway lead to more prolonged and profound T-cell activation.

Indications for all five of the approved PD-1/PD-L1 inhibitors include advanced/metastatic urothelial carcinoma. Supporting data for the approvals came from a wide range of clinical trials, encompassing phase I, II, and III investigations.

Dr. Li reviewed data for the two newest PD-1/PD-L1 inhibitors from clinical trials involving patients with disease progression after platinum-based chemotherapy. Avelumab was evaluated in a phase Ib trial involving 44 patients (Apolo et al., 2017). The trial results showed a median overall survival of 13.7 months, 1-year survival of 54%, and median PFS of 2.9 months. A phase I/II trial of durvalumab involved 191 patients and demonstrated a median survival of 18.2 months, 1-year survival of 55%, and median PFS of 1.5 months (Powles et al., 2017).

Atezolizumab (Tecentriq), nivolumab (Opdivo), and pembrolizumab (Keytruda) were evaluated in phase I to III trials involving 86 to 542 patients with advanced/metastatic urothelial cancer. Results of those trials showed a median overall survival of 7.9 to 10.3 months, 1-year survival of 36% to 46%, and median PFS of 2.1 to 2.8 months (Bellmunt et al., 2017; Rosenberg et al., 2016; Sharma et al., 2016).

"When you look at the studies with avelumab and durvalumab, they were pretty much in line with the other studies," said Dr. Li. "The issue here is the different studies’ designs, and you can’t really make many inferences in terms of which one is better at this point in time because of that."

Comparability of the PD-1/PD-L1 inhibitors extends to their safety profile. Fatigue occurs commonly in patients treated with any of the agents. Infusion site reactions and hypertension occur more often with avelumab. Arthralgia, diarrhea, rash, decreased appetite, and dyspnea occur to varying degrees across the drug class.

With respect to special monitoring for immune-related adverse events, the principal concerns are pneumonitis, hepatitis, and colitis. Patients treated with avelumab require premedication with acetaminophen and an antihistamine to minimize the risk of infusion reactions for at least the first four infusions, said Dr. Li.

Where overlapping indications exist, the five available PD-1/PD-L1 inhibitors will compete for a therapeutic role. Physician and patient preference, insurance coverage, and clinical judgment regarding toxicities will often influence the choice of drug when more than one option is available, said Dr. Li.

## TYROSINE KINASE INHIBITORS

The newest TKIs are brigatinib (Alunbrig) and neratinib (Nerlynx). Brigatinib received FDA approval for patients with ALK-positive NSCLC that has progressed on crizotinib (Xalkori). Neratinib’s approved indication is for extended adjuvant treatment of early HER2-positive breast cancer after adjuvant trastuzumab (Herceptin).

Brigatinib is a multitargeted TKI, inhibiting *ROS1*, *IGF-1*, *FLT3*, *EGFR* deletion, and point mutations, in addition to *ALK*. The drug joins a list of more than a half-dozen TKIs that target *EGFR*, said Dr. Li.

Brigatinib was evaluated in one phase I/II trial and a phase II trial involving patients with ALK-positive NSCLC that proved refractory to crizotinib. The phase I/II trial had a primary outcome of objective response, which was 72% in 42 evaluable patients (Gettinger et al., 2016). The phase II trial evaluated two different doses of brigatinib in a total of 222 patients (Kim et al., 2017). The results showed response rates of 45% and 54% with the lower and higher doses, respectively, median PFS of 9.2 and 12.9 months, and 1-year survival of 70.6% and 79.5%.

The principal support for neratinib’s approval came from a phase III, randomized, placebo-controlled trial involving 1,420 patients with early HER2-positive breast cancer (Chan et al., 2016). All the patients had completed a standard course of adjuvant trastuzumab and were randomized to an additional 12 months of treatment with neratinib or placebo.

The trial had a primary endpoint of invasive disease-free survival (IDFS) at 2 years. The results showed that 70 qualifying events occurred in the neratinib arm vs. 109 in the placebo group (*p* = .0091). The 2-year IDFS was 93.9% with neratinib and 91.6% with placebo.

"If you really look at things, we’re talking about preventing 39 events out of 1,420 patients," said Dr. Li. "That’s a number needed to treat of 36. What we’re talking about is treating 36 patients to prevent one event. We’re committing 36 patients to a therapy that lasts a year and only one of them will actually receive a benefit.

"I’m not saying that this doesn’t work, but we have to think a little bit about who would be the best patient to actually get this and reducing that number needed to treat. That’s going to be an issue in terms of how do you select out who actually should receive this product."

The principal toxicity with neratinib is diarrhea, which occurs in almost all patients, consistent with EGFR inhibition, and reached grade 3/4 severity in about 40% of patients. Other gastrointestinal effects, such as vomiting and abdominal pain, affect a substantial proportion of patients, and about 20% of patients developed rash. Aside from diarrhea, grade 3/4 adverse events are fairly uncommon.

Because it inhibits multiple tyrosine kinases, brigatinib has a broad range of adverse events, particularly laboratory events such as elevated liver enzymes, hyperglycemia, amylase, lipase, activated partial thromboplastin time, and anemia. Additionally, 15% to 30% of patients develop diarrhea, vomiting, abdominal pain, cough, dyspnea, arthralgia, and decreased appetite. Grade 3/4 adverse events are uncommon.

Patients treated with brigatinib warrant special monitoring for interstitial lung disease, hypertension and other cardiovascular toxicities, visual disturbances, pancreatic enzyme elevation, and hyperglycemia. With neratinib, the principal concern is diarrhea.

"Brigatinib represents an advancement in the treatment of resistant and refractory ALK-positive NSCLC, and use of this agent for specific mutations may represent the future of precision medicine in NSCLC," Dr. Li said in conclusion.

"Neratinib represents another option for early treatment of HER2-positive breast cancer. Standard of care currently includes pertuzumab (Perjeta) in the adjuvant setting, and the studies did not include this agent. So neratinib would be for patients who did not get or did not qualify for pertuzumab."
